# A rare association between a dermoid cyst and arachnoid cyst of the cerebellopontine angle: a case report

**DOI:** 10.11604/pamj.2021.40.125.32040

**Published:** 2021-11-01

**Authors:** Zoubeyr Abbou, Rania Djennati, Zeyad Khalil

**Affiliations:** 1 Department of Neurosurgery, Faculty of Medicine, Abou Bekr Belkaid University, Tlemcen, Algeria,; 2 Royal Preston Hospital, Manchester, United Kingdom,; 3 Manchester Foundation Trust-South, Manchester, United Kingdom

**Keywords:** Arachnoid cyst, dermoid cyst, cerebellopontine angle, child, case report

## Abstract

Supratentorial arachnoid cysts are usually asymptomatic and may be discovered by chance at autopsy; however, infratentorial arachnoid cysts, which correspond to liquid forms enclosed by an arachnoid sheet but whose pathogenesis is unknown, might cause symptoms. They don't need to be treated if they're asymptomatic and were discovered by chance. A variety of neurological symptoms can be present depending on their localization. Intracranial dermoid cysts are uncommon tumours that develop from ectopic epithelial cells in the brain. They are benign, slow-growing, and rarely rupture. The association between the two diseases is extremely rare and when it is present may suggest the existence of a common factor. We present a unique case of a young girl who developed headache and ataxia as a result of an intracranial infratentorial dermoid cyst and an arachnoid cyst of the cerebellopontine angle. Complete removal of the dermoid cyst and drainage of the cyst leads to a full recovery. Dermoid and arachnoid cyst are two pathologies with a possible common embryogenic factor, early surgery can give a better outcome in the long term.

## Introduction

In 1855, Quain presented the first description of a supratentorial cerebral arachnoid cyst. Arachnoid cysts in the posterior fossa were first reported by Maunsell in 1889. Under the term “chronic cystic arachnoiditis”. Craig attributed the clinical picture to an inflammatory reaction of the leptomeninges. Many hypotheses offered to explain the development of these cysts have included congenital malformation, infection and trauma. Arachnoid cysts have been reported in connection with tumours in the posterior fossa, and are thought to be caused by a transudate [1]. The arachnoid cyst is a cystic formation with an arachnoid wall and the contents formed by the cerebrospinal fluid (CSF). Its congenital origin is accepted by most authors and results from aberrant development of the arachnoid tissue [1]. Symptomatic arachnoid cysts represent 1% of intracerebral neoforms, the child being affected in 60% to 90% of cases [2].

Dermoid cysts are a rare benign tumour of the central nervous system and accounts for 0.1 to 0.7% of all intracranial processes. It is formed by a thick wall, lined by a keratinizing squamous epithelium and its content is composed of dermal elements, hair, teeth and sebaceous glands. It is a congenital tumour due to a developmental abnormality between the third and fifth week of embryonic life, its clinical manifestation is mainly in young adolescents [3]. The association between an arachnoid cyst and a dermoid cyst is very rare. We found only one case described in the literature, reported by Chhang WH in 1989 [4]; where he describe an arachnoid cyst of the middle fossa associated with a suprasellar dermoid cyst. In this case, we report a rare association between an arachnoid cyst of a cerebellopontine angle with a vermian dermoid cyst.

## Patient and observation

**Patient information:** we report a case of a 3-year-old child, born of a completed pregnancy, fourth of four children, she has been suffering from mild intensity headache for the past 6 months treated by simple analgesics.

**Figure 1:**
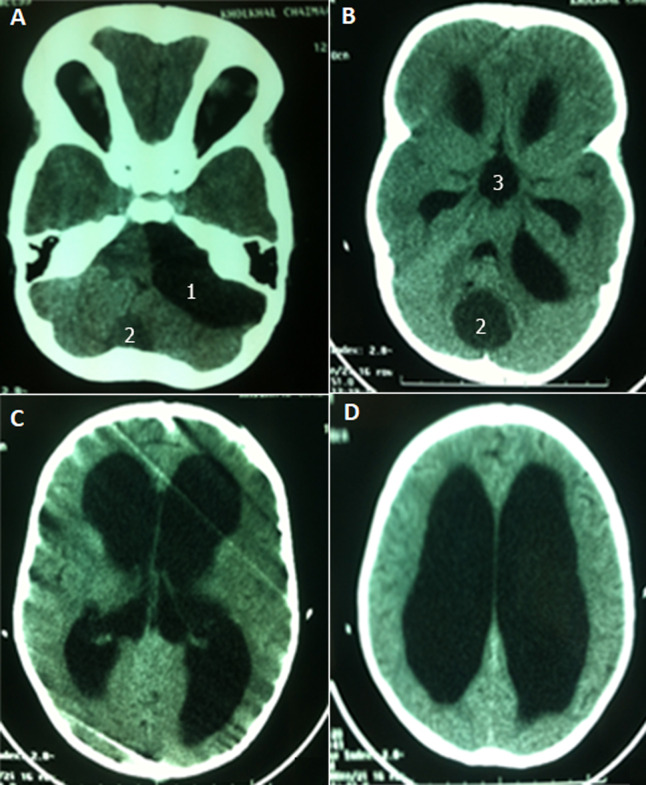
(A,B,C,D) axial non contrast CT scan showing CPA arachnoid cyst of the cerebellopontine angle (1), a vermian dermoid cyst (2) and significant dilatation of the third ventricle and lateral ventricles

**Clinical and radiological findings:** our patient has been admitted to A&E for headache, nausea, vomiting and loss of consciousness. On examination, she had a GCS 12, slight macrocrania, static cerebellar syndrome makes of an eccentric gait. Computed tomography (CT) scan visualized two cystic formations of the posterior cerebral fossa with active tri-ventricular hydrocephalus (Figure 1).

**Figure 2:**
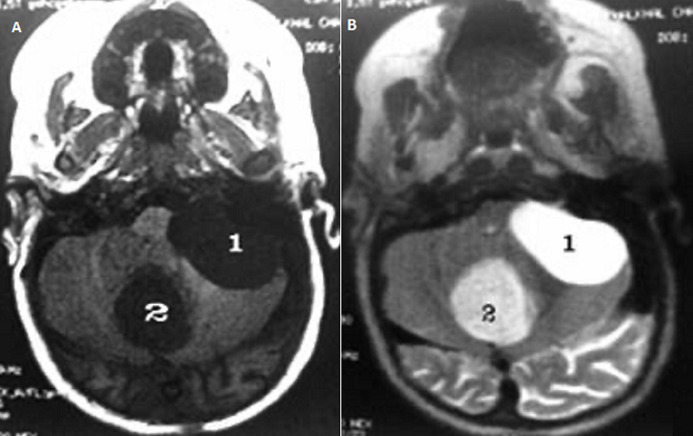
A) cerebral MRI in axial section performed in T1; B) cerebral MRI in axial section performed in T2 mode showing the arachnoid cyst of the left cerebellopontine angle (1) in hypo-signal T1 (A) and in hyper-signal T2 (B) and the vermian dermoid cyst (2) in hypo-signal T1 (A) and slightly in hyper-signal T2 (B)

**Figure 3:**
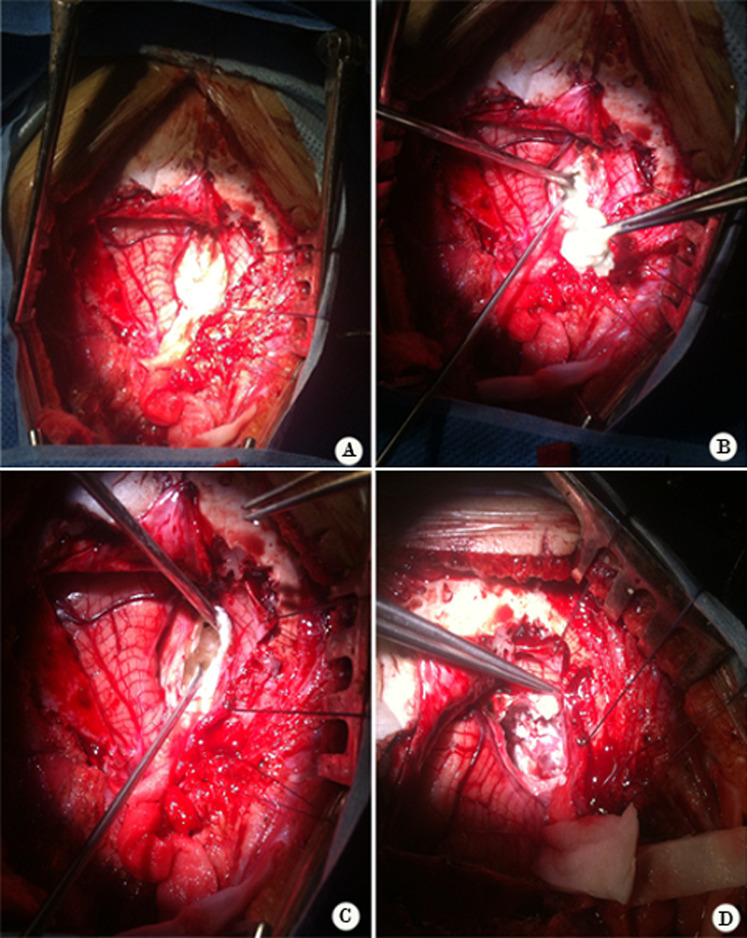
(A,B,C,D) per-operative view of the dermoid cyst being completely removed using a median sub-occipital approach

**Figure 4:**
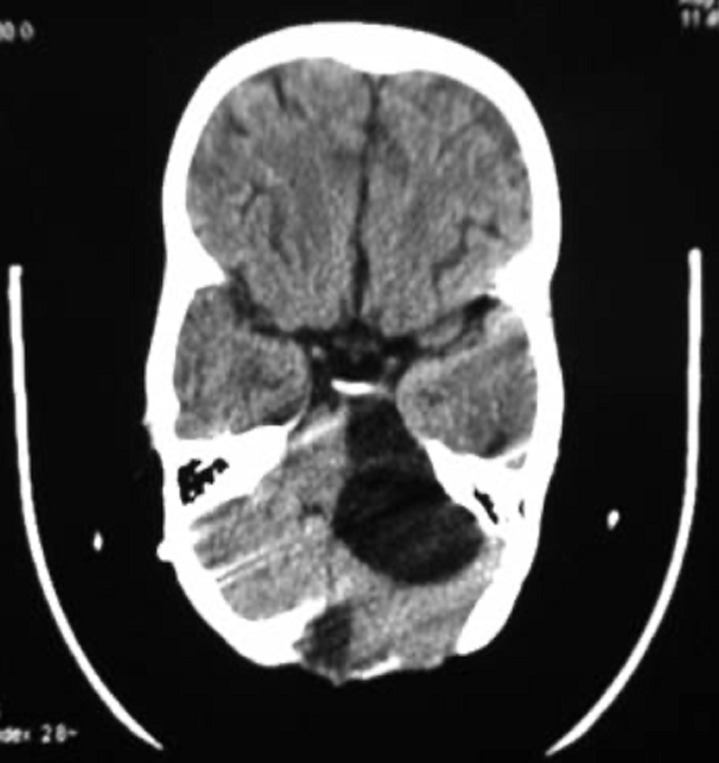
postoperative cerebral CT scan which allows to visualizing the total excision of the dermoid cyst via the median sub-occipital approach, as well as the arachnoid cyst of the cerebellopontine angle not yet operated

**Figure 5:**
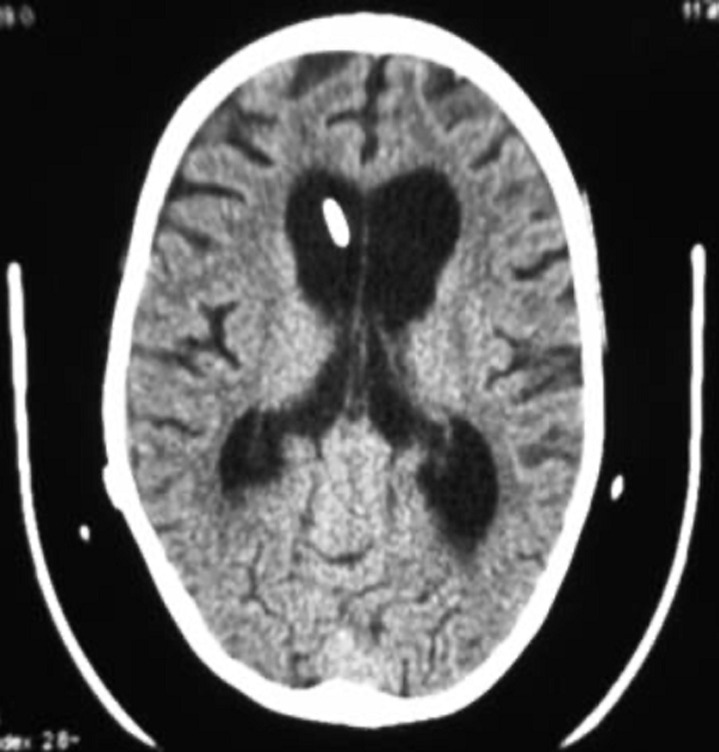
the cerebral CT scan also shows the right frontal ventriculoperitoneal shunt valve in place, with normal-sized ventricles

**Therapeutic interventions and follow-up:** a ventriculoperitoneal shunt has been placed in an emergency which allowed normalization of consciousness and cerebral magnetic resonance imaging (MRI) was performed later on and has confirmed the arachnoid cyst of the cerebellopontine angle (CPA) and vermian dermoid cyst which compress the brainstem anteriorly (Figure 2). A second surgical procedure was performed to remove the vermian dermoid cyst, through a medial sub-occipital craniectomy (Figure 3) and performing a post-operative CT scan showing a total removal of the dermoid cyst and the perfect placement of the ventriculoperitoneal valve (Figure 4, Figure 5).

**Figure 6:**
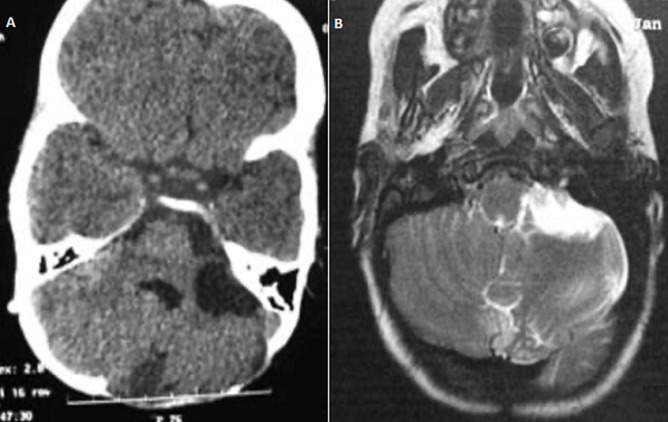
A) cerebral CT scan; B) cerebral MRI show a collapse of the arachnoid cyst from the cerebellopontine angle and total excision of the vermian dermoid cyst

After the removal of the dermoid cyst, the cerebellar syndrome disappeared completely, however, three months later, the young girl returned with new symptomatology made of lateral deviation of the eyes towards the right side, due to the compression of the left arachnoid cyst of the CPA on the brainstem. The young girl has another operation for a marsupialization of the arachnoid cyst of the CPA by a retro-sigmoid craniotomy, with partial resection of the external wall and establish a communication with the cisterna magna and fenestration of the inner wall, thus communicating with the pre-pontine and pre-bulbar cisterns. The postoperative observation showed the disappearance of the lateral deviation of the eyes and six months CT scan showed the decrease in the size of the CPA arachnoid cyst (Figure 6).

## Discussion

Several theories have been proposed to explain the arachnoid cyst's etiopathogenesis, but the embryological theory is the most widely accepted. The subarachnoid space between the pia mater and the arachnoid is established during hydraulic dissection by the CSF produced by the primary ventricular system during the fourth month of intrauterine life. The arachnoid is not well-differentiated at this stage of embryonic development, and hydraulic dissection between the two layers of the arachnoid might result in an arachnoid cyst [1,2,4]. The embryological aetiology of the dermoid cyst is also established. It is caused by an aberration in embryonic development between the fifth and sixth weeks of pregnancy, which is caused by the inclusion of ectodermal elements in the neural tube as a result of the latter's inability to close [3,4].

In this case, two anomalies of embryonic development were found in the same patient. As a result, this instance raises the question of whether these two developmental defects share an etiological cause. The arachnoid cyst accounts for 1% of intracranial neoformations, and the sylvic localization is the most common, accounting for 50% of instances, whereas only 8% are found at the cerebellopontine angle. The dermoid cyst accounts for 0.1 to 0.7% of intracranial neoformations and is usually seen on or around the midline [2,4].

In the case of a dermoid cyst and arachnoid cyst association described by Chhang WH in 1989 [4], just like the case we are reporting here, it is to be noted that there is a relation between the dermoid cyst and the arachnoid cyst; where he described the arachnoid cyst is in the middle fossa and the dermoid cyst is the suprasellar region. This similarity, in our opinion, supports the existence of a single etiological component, which is responsible for the embryogenesis aberration that causes the dermoid cyst and the arachnoid cyst [4]. Symptomatic cerebellopontine angle arachnoid cyst is usually expressed by signs of intracranial hypertension, cerebellar symptomatology and cranial nerve damage: V (trigeminal neuralgia), VI (diplopia), VII (hemiface spasm or facial palsy), VIII (hearing loss, tinnitus and vertigo). For vermian dermoid cyst, the clinical manifestation is usually intracranial hypertension syndrome and cerebellar syndrome [5]. Intracranial pressure syndrome, right lateral deviation of the eyes, and an eccentric gait were all present in our patient. CT scan and MRI are the gold standards in confirming and differentiating between the two pathologies (Table 1). Dermoid cysts and arachnoid cyst appear most of the time hypodense in CT scan with or without contrast. On MRI the dermoid cysts are hyperintense T1 due to high lipid content and heterogenous on T2 due to the mixed components in the cyst (cartilage, bones and calcification). The MRI FLAIR is a key to differentiate between the fat (hyperintense) and liquid (hypointense). It is worth mentioning that there is no contrast enhancement for dermoid and arachnoid cysts on MRI [6,7].

**Table 1:** radiologic findings between dermoid cyst and arachnoid cyst

Investigation		Dermoid cyst	Arachnoid cyst	Contrast enhancement	
				Dermoid cyst	Arachnoid cyst
**CT scan**		Mixed density	Hypodense	**-**	**-**
		Hypodense			
**MRI**	**T1**	Hyperintense	Hypointense	**-**	**-**
	**T2**	Hypo or hyperintense	Hyperintense		
	**DWI**	Hyperintense			
	**ADC**	Isointense			
	**FLAIR**	Fat: Hyperintense			
		Bone, cartilage: hypointense			

CT: computed tomography; MRI: magnetic resonance imaging; DWI: diffusion-weighted imaging; ADC: apparent diffusion coefficient; FLAIR: fluid attenuated inversion recovery

Three surgical procedures for arachnoid cysts are described: excision of the cystic wall (open surgery), fenestration of the wall (endoscopy and microscopy), and cysto-peritoneal and cysto-subdural shunts [8]. The presence of intra-cystic septa, fine nerve structures, and a high rate of recurrence in the event of fenestration all point to excision of the cystic wall with a microscope in the case of an arachnoid cyst of the cerebellopontine angle [9,10]. The reduction in the size of an infratentorial arachnoid cyst can only be achieved after a significant resection of its wall under the microscope. Partial resection of the external wall was performed in our patient, along with fenestration of the internal wall. The dermoid cyst has a thick cystic wall made of stratified squamous epithelium, with dermal components, fat cells, hair, and sebaceous glands as contents. To decrease the danger of recurrence, total microsurgical excision of the dermoid cyst is recommended. Capsule fragments may, however, be left in place in front of a cyst wall that is firmly adhered to a sensitive nerve structure after being carefully coagulated. The evolution of these fragments is slow; thus, no extra treatment is required immediately. Our patient had a vermian dermoid cyst that was unrelated to the floor of the fourth ventricle, allowing for its complete removal [11].

The therapeutic strategy used in this patient included ventriculoperitoneal bypass as the first step, dermal cyst surgery as the second, and arachnoid cyst surgery as the third step. The unconscious state due to active hydrocephalus justified the emergency ventriculoperitoneal bypass, the static cerebellar syndrome due to the dermoid cyst justified the scheduling of a cyst surgery in the second place, and the second appearance of a lateral deviation of the eyes, after the two previous surgeries, due to arachnoid cyst compression of the brainstem, justified the scheduling of a cyst surgery in the third place. This therapeutic timeline was based on clinical manifestations when the arachnoid cyst was bigger and compressing the brainstem radiographically. Wasn't it more prudent to begin with the treatment of the arachnoid cyst, which, due to its size and compression of the brainstem, posed a serious risk, notwithstanding the dermoid cyst's clinical manifestation of a simple eccentric gait?

## Conclusion

The arachnidian cyst, like the dermoid cyst, is a rare pathology, and the relationship between these two cystic structures at the level of the posterior cerebral fossa is particularly exceptional. The proximity of these two lesions raises the idea of a similar factor causing embryonic development issues, both at the level of the neural tube, where the dermoid cyst forms, and at the level of the sub-arachnidian region, where the arachnoidian cyst forms.
